# Red blood cell extracellular vesicles: new frontiers in hematological biomarker discovery

**DOI:** 10.3389/fmed.2025.1644077

**Published:** 2025-08-18

**Authors:** Urja Joshi, Linz-Buoy George, Hyacinth Highland

**Affiliations:** ^1^Department of Biochemistry, School of Sciences, Gujarat University, Ahmedabad, India; ^2^Department of Zoology, Biomedical Technology, Human Genetics & Wildlife Biology and Conservation, School of Sciences, Gujarat University, Ahmedabad, India

**Keywords:** extracellular vesicles (EVs), red blood cells (RBCs), hematological biomarkers, liquid biopsy, cell to cell communications

## Abstract

Extracellular vesicles (EVs) offer promising opportunities in hematology for improved diagnostics, prognostics, and therapeutics, making them valuable tools in the molecular landscape. EVs derived from red blood cells (RBCs) are the primary source of EVs in the bloodstream. They perform several critical biological and physiological functions, such as facilitating intercellular communication and transferring biomolecules like DNA, RNA, and proteins. Hence, in this review, we aim to explore RBC-derived EVs and their potential as a diagnostic tool for their clinical relevance and associated biomarkers in hematology. Furthermore, we emphasized their crucial role in both physiology and disease. RBC-EVs are found to play a role in vascular damage, inflammation, and coagulopathy in several pathophysiological conditions, potentially influencing the progression of certain diseases. They also served as indicators for numerous conditions, including hereditary hematologic abnormalities, diabetes, and cardiovascular diseases. Hence, their importance lies in their ability to reflect and influence red cell health, immune responses, and systemic disease states as accessible, non-invasive indicators. Also, their composition mirrors the physiological or pathological state of RBCs and holds promise for both diagnostics and therapeutics.

## 1 Introduction

Extracellular vesicles (EVs) are gaining importance in hematology as non-invasive biomarkers for early diagnosis and disease monitoring, providing insights into disease mechanisms, as they carry disease-specific proteins, lipids, and nucleic acids (1). The subtypes of EVs that are being increasingly explored in hematology include microvesicles (100–1000 nm), exosomes (30–200 nm), and apoptotic bodies (50–5000 nm) in diameter. These subtypes vary depending on their biogenesis, release pathways, size, content, and functions (2). RBCs outnumber all other blood cells, comprising ∼84% of all cells in the human body and over 99% of all blood cells by number. This sheer numerical dominance means that even if a small fraction of RBCs release vesicles, the absolute amount of RBC-EVs will be substantial, hence, RBC-derived EVs dominate the blood EV population (3). Additionally, RBCs naturally shed membranes due to aging or stress, lacking organelles for repair and protein synthesis themselves. They release damaged components through EV release, preserving flexibility and function (4, 5). RBCs circulate for ∼120 days. As they age, membrane remodeling increases, and senescent RBCs release microvesicles and exosomes to discard damaged components. This vesiculation is one of the early signals for RBC clearance by macrophages (6). RBC-EVs circulate in plasma, transporting intact biomolecules; their concentrations and properties can be employed to differentiate among distinct disease stages and phases; thus, they act as “liquid biopsy” readouts of RBC health (7).

**FIGURE 1:**
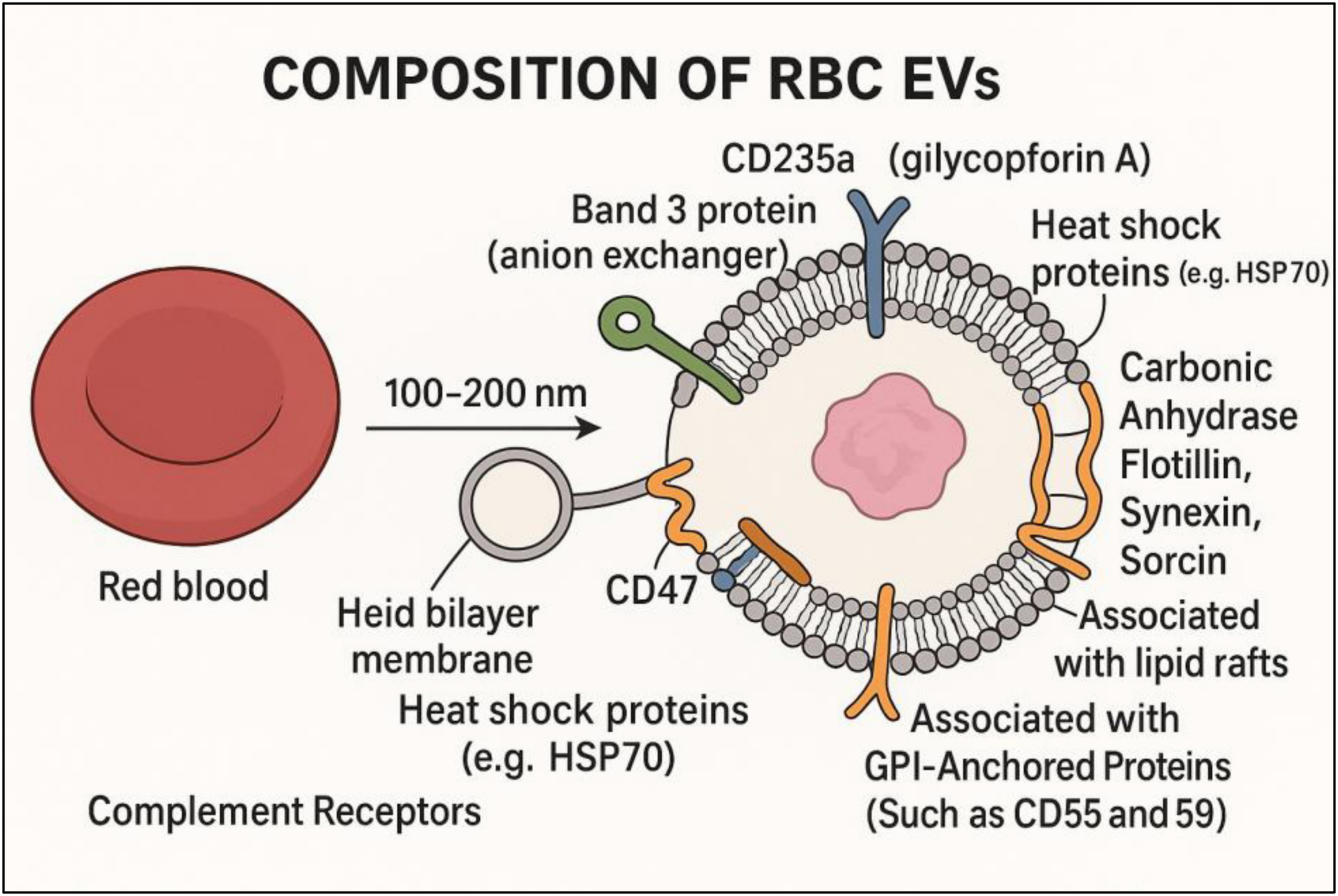
Composition of RBC-derived EVs (RBC-EVs).

As shown in Figure 1, RBC-EVs are defined by surface molecules like tetraspanins (CD9, CD63, CD81), specific markers (e.g., glycophorins/band 3, CD235a) (8), and phosphatidylserine exposure (9). They are counted using flow cytometry (CD235a+ or Annexin V + microparticles) and profiled using proteomics or RNA assays (10), discussed in Table 1. These surface molecules on EVs aid in identifying and binding to specific recipient cells, ensuring effective delivery of EV contents (proteins, RNAs, lipids), similar to viruses or hormones (3). Integrins facilitate organotropism and direct EVs to specific tissues like the liver, brain, lungs, etc. While a few Heparan sulfate proteoglycans (HSPGs) are involved in initial EV binding to recipient cell surfaces (11). EVs also carry MHC molecules, cytokines, or ligands that activate or suppress immune responses, aiding communication between immune and non-immune cells, and can induce apoptosis or immune suppression (12).

**TABLE 1:** Key contents of red blood cell-derived extracellular vesicles (RBC-EVs).

Component type	Details
Proteins	Hemoglobin (HbA, HbS) - Band 3 protein (anion exchanger) - Spectrin, Ankyrin, Actin (cytoskeletal) - Heat shock proteins (e.g., HSP70) - Carbonic anhydrase, Stomatin, Flotillin, Synexin, Sorcin - associated with the lipid rafts (3). Complement Receptors and GPI-anchored proteins such as CD55 and CD59 protect cells from complement-mediated lysis (5).
Lipids	Phosphatidylserine (PS) (externalized, procoagulant), phosphatidylethanolamine (PE), phosphatidic acid (PA), Cholesterol, sphingomyelin - Oxidized lipids (in stress/aging) (18, 19).
microRNAs (miRNAs)	miR-451a (most abundant in RBCs), miR-144, miR-486, miR-92a (involved in erythropoiesis and red cell stress response) (20), miR-125 b-5p, miR-4454, and miR-451a (expressed in RBC-EVs released from the stored RBC) (21).
Other RNAs	Some studies report small non-coding RNAs and tRNA fragments (13).
Oxidative markers	Oxidized proteins, lipid peroxides, and advanced glycation end-products (AGEs) (3).
Surface markers	CD235a (Glycophorin A), tetraspanins (CD9, CD63, CD81), CD71 (transferrin receptor, early erythroid cells), Annexin V, PS (8–10).

RBC-EVs contain specific biomolecules reflecting parent RBC composition and functional state, providing informative content in disease contexts due to their simpler nature without nuclei and organelles (13). Their EV production is an essential mechanism of homeostasis and is amplified in many disease states, making them a major source of circulating EVs. Studies show that RBC-EVs’ levels or contents change in disease, raising diagnostic interest (14, 15). Conditions like oxidative stress, infection, inflammation, hypoxia, hemolysis, and even exercise increase RBC-EV shedding. Diseases such as sickle cell anemia, thalassemia, malaria, and diabetes are known to cause RBCs to release more EVs than normal (16). Also, diseases such as sickle cell anemia, thalassemia, malaria, and diabetes are known to cause RBCs to release more EVs than normal (17).

## 2 Mechanistic insights into the pathogenicity of RBC-EVs

RBC-EVs are not inert byproducts, but bioactive mediators in both physiological processes and pathological conditions. Despite being enucleated, RBCs interact with their environment. Changes in systemic inflammation, oxidative stress, pH changes, calcium influx, or infections can lead to modifications in RBCs, and these changes get passed to their EVs (22). For instance, RBC EVs carry disease-specific cargo from patients with certain diseases, such as oxidative stress markers (e.g., 4-HNE, ROS-modified proteins), pro-coagulant factors (e.g., phosphatidylserine, tissue factor), and inflammatory signals (e.g., complement proteins in autoimmune diseases), regulatory miRNAs (e.g., miR-451, miR-144, etc.) (23). These can be transferred to endothelial cells, macrophages, and even neuronal cells, altering gene expression, inflammatory signaling, and cell survival pathways. In neurovascular or cardiovascular disease, RBC-EV cargo may exacerbate inflammation, cell death, or immune dysregulation. Thus, RBC EVs can serve as sentinels of broader pathophysiology beyond RBCs alone (23). They can also be readily extracted from biofluids like blood, urine, etc., and are considerably stable under various conditions (24). They offer a “liquid biopsy” of red blood cell functionality and overall health of the body (7). However, RBC-EVs’ heterogeneity is a considerable challenge in diagnostic applications (25). They are typically identified by the expression of CD9, CD63, and CD81, glycophorin A (CD235a) and B, displayed in Figure 1. Additional protein indicators commonly linked to exosomes include flotillin, TSG101, Alix, HSP60, HSP70, HSPA5, CCT2, and HSP90 (26). RBC-EVs are recognized as active participants, particularly in vascular and inflammatory diseases, due to their pro-inflammatory, pro-coagulant, and redox-active cargoes (22). PS is a key component of the integral membrane of RBCs, which can be exposed to the outer surface during RBC aging, stress (e.g., in sickle cell disease, malaria, sepsis), or EV formation. PS becomes externalized on the outer leaflet of EVs. This exposed PS acts as a catalytic surface for the tenase and prothrombinase complexes (factor Xa + factor Va), significantly accelerating fibrin clot formation, thrombin generation. Increased PS + RBC-EVs are observed in sickle cell illness, sepsis, and cardiovascular disease, all linked to thrombo-inflammation (18, 19, 27). Additionally, in Beta-thalassemia, chronic hemolysis increases PS + EVs, enhancing thrombotic risk (28).

RBC-EVs often contain cell-free hemoglobin or heme, which can enter endothelial cells via scavenger receptors (e.g., CD91, TLR4), activate TLR4 signaling, promoting pro-inflammatory gene expression (IL-6, VCAM-1, ICAM-1, E-selectin), and generate reactive oxygen species (ROS) via Fenton chemistry. These lead to endothelial dysfunction, leukocyte adhesion, and vascular permeability in SCD, sepsis, and transfusion-related complications (29, 30). As well, cell-free Hb in RBC-EVs scavenges and binds to nitric oxide (NO) with high affinity, Hb + NO → MetHb + nitrate. This scavenging reduces bioavailable NO, a key vasodilator and anti-platelet mediator (31). Which leads to vasoconstriction, platelet activation, and endothelial dysfunction and amplifies complications like pulmonary hypertension, especially in chronic hemolytic states (32, 33). Hence, Hb-loaded EVs contribute to vaso-occlusive crises and pulmonary hypertension, with elevated hemolysis observed in sickle cell, malaria, PNH, etc. (34). Mantel et al. also reported that in malaria, parasite-altered RBC-EVs increase endothelial activation and brain microvascular dysfunction (35). Furthermore, the CD47 signal on EVs interacts with signal regulatory protein alpha (SIRPα) on the macrophage surface, inhibiting phagocytosis and immune system activation (36). While several other RBC surface proteins restrict the interactions with the complement system are C8 binding protein (C8bp) (37), homologous restriction protein (HRP) (38), decay accelerating factor (DAF) (39), membrane cofactor protein (MCP), complement receptor 1 (CR1), and CD59 (40). Increased oxidative stress enhances Band 3 phosphorylation, clustering, and dissociation from other cytoskeletal proteins, while also initiating the elimination of aged red blood cells, phagocytosis, and complement activation (41).

Some RBC-EVs express adhesion molecules such as CD36, ICAM-4, which can bind to plasma proteins like fibrinogen, promoting EV-endothelium adhesion, EV-platelet or EV-leukocyte interactions. This is important in diseases with systemic inflammation (e.g., malaria, COVID-19, sepsis) because it facilitates leukocyte recruitment, platelet aggregation (5). Hence, an altered protein or lipid composition of RBC-EVs may indicate oxidative damage, inflammation, or disease conditions, aiding in the diagnosis and monitoring of illnesses (8), discussed in Table 2.

**TABLE 2:** Pathological signatures of RBC-EVs: key biomarkers in health and disease.

Biomarker (RBC- EV)	Condition/context	Diagnostic performance (sensitivity/specificity)	References
PS + , CD235a + EVs; miR-451a, hemoglobin	Sickle cell disease (hemolysis indicator)	Elevated in SCD; correlates with LDH, heme. (No ROC reported)	(42, 43)
EVs + α- and β-globin chains + HSP70 Lower levels of free Hb-scavenging plasma proteins and immunoglobulin chains (HbE/beta-thalassemic patients)	Thalassemia	High EV count due to ineffective erythropoiesis and increased turnover	(28)
PS + EVs; MPs, oxidized proteins	Glucose 6 Phosphate Deficiency (G6PD) severity	4 × higher in severe G6PD vs moderate. (ROC not yet defined), Oxidative damage triggers EV shedding	(17, 44)
PS + , MPs + EVs	Pulmonary arterial hypertension (PAH)	PS^∧^ + RBC-EV increased vs controls (no ROC).	(45)
PS + , MPs + EVs	Systemic lupus erythematosus (SLE)	Total and PS^∧^ + RBC-EV↑; high PS^∧^ + level identifies past thrombosis.	(46)
PS + , MPs + EVs	Glomerular hematuria (GH) (renal bleed)	Urinary RBC-EVs higher in glomerular vs non-glomerular hematuria.	(47)
PS + , MPs + EVs + altered lipid/protein cargo)	Type 2 diabetes (with complications)	↑RBC-EV count (26 vs 9/μL); correlates with fasting glucose. Hyperglycemia causes membrane glycation, oxidative stress	(48)
Exosomal miR-451a and let-7i-5p	Sickle cell disease vs healthy	AUC≈0.83–0.84 distinguishing HbSS/SC vs HbAA.	(43)
MPs + EVs, miR-451a, let-7i-5p, parasite-derived peptides, plasmodium gDNA in ring stage iRBCs EVs, *Pf*EMP1, Knob associated proteins, lipids such as PS, PI, PC	Malaria susceptibility (Hb genotype)	Levels vary with Hb genotype; they correlate with parasite growth. Infected RBCs release EVs that carry parasite content	(49–51)
PS + , MPs + EVs, annexin V + , tissue factor	Cardiovascular disease	Pro-coagulant EVs may contribute to thrombosis	(52)
EV-associated hemoglobin/heme	Hemolytic anemia, transfusion	Reflects RBC breakdown; potential marker of intravascular hemolysis (no ROC data).	(53)
α-synuclein in RBC- EVs	Parkinson’s disease (PD)	Elevated in PD patients’ RBC-EVs; diagnostic stats pending. RBC-EVs carry α-synuclein to the CNS and peripheral tissues	(54)
CD235a (glycophorin A) on the EV surface	General RBC-EV identification and a fold increase during *P. falciparum* infection	Used to quantify RBC-EVs specifically (flow cytometry).	(8)
EVs + oxidized lipids, band 3 clustering, increased externalization of PS on RBC membranes	Aging/senescence	Enhanced EV production during aging	(55, 56)
Transient PS + , CD235a + EV, signaling molecules	Exercise/hypoxia	Temporary rise in EV levels due to mechanical/oxidative stress, induced vesiculation	(57, 58)

Table 2 summarizes selected red cell EV–derived biomarkers and their reported disease associations.

## 3 Biomarkers in inflammation, vascular damage, coagulation, and infection

RBC EVs provide a real-time snapshot of ongoing physiological or pathological processes. Their surface markers and cargo drive thrombo-inflammation through defined pathways, such as coagulation amplification, endothelial activation, NO depletion, and gene regulation (59). RBC-EVs can directly impair the function of endothelial cells, the cells via transferring the cargo molecules, changing permeability, activating immune cells, leukocyte/platelet adhesion, etc (59). For example, in conditions like type 2 diabetes, RBC-EVs can be taken up by endothelial cells and transfer pro-oxidant enzymes like Arg1, leading to impaired endothelium-dependent relaxation and promoting endothelial dysfunction (60). EVs, including those derived from RBCs, can influence vascular remodeling, a process involved in the development of various vascular diseases. They can affect oxidative stress, inflammation, calcification, and lipid plaque formation, all of which contribute to vascular remodeling (60, 61).

RBC-EVs can promote the production of pro-inflammatory cytokines like TNF-α, IL-6, and IL-1β. They can also promote the activation of immune cells like monocytes and neutrophils, leading to increased inflammation and adhesion to the endothelium (19). In sepsis, RBC-EVs can exacerbate the inflammatory response and reduce survival rates. In arthritis, RBC-EVs are involved in the inflammatory process in the inflammatory fluid of patients with arthritis, serving as a source of the lipid mediator LPA (62). Whereas, in malaria, RBCs infected with the malaria parasite Plasmodium falciparum release EVs that contribute to the local and systemic production of pro-inflammatory cytokines and chemokines, leading to vascular dysfunction and increasing the EC permeability by downregulating caveolin-1 and activating transcription factor 2 (63).

RBC-EVs also possess procoagulant properties, contributing to the activation of the coagulation cascade. This action pertains to the exposure of phosphatidylserine (PS) on their surface, which offers a negatively charged interface for the formation and activation of coagulation complexes. RBC-EVs can trigger thrombin production in an FXII-dependent way, independent of tissue factor (TF) (64). Therefore, increased levels of procoagulant RBC-EVs are observed in conditions like sudden nocturnal hemoglobinuria (PNH) and hemolytic disease, contributing to increased thrombotic and hypercoagulable states (64). It has also been found that RBC-EVs present in stored RBC products have been implicated in transfusion-related adverse effects, including a significant procoagulant effect that may worsen the condition of patients with a hypercoagulable state (65).

RBC-EVs increase in many inflammatory and infectious scenarios, indicating the immunological activation of RBCs. It has been discovered that they also harbor viral proteins, including those from respiratory viruses and SARS-CoV-2, before the production of antibodies (66). In severe dengue virus infection, dengue-infected cells provoke extensive apoptosis of red blood cells and the generation of extracellular vesicles. A clinical study found a significant correlation between higher RBC-EV levels and dengue severity, suggesting RBC-EV count can determine disease stage and be a prognostic diagnostic tool for dengue (67).

In malaria, RBC-EVs from infected cells carry parasite antigens and modulate immunity (68). Additionally, Nantakomol et al. reported that the EV secretion of P. *falciparum*-iRBCs was tenfold more than that of non-iRBCs and escalated with parasite maturation (69). The study found that malaria-infected RBCs release EVs with various human RNAs, including miRNAs and tRNA, Y-RNAs, vault RNAs, snoRNAs, piRNAs, and plasmodium RNAs. These RNAs can be transferred to human endothelial cells, potentially regulating gene expression and altering barrier properties. Additionally, plasmodium proteins, including ring-infected erythrocyte surface antigen, have proinflammatory activity and contribute to malaria pathology (63, 70). Malaria-infected RBC-EVs activate natural killer (NK) cells via MAD5 and monocytes, leading to immune elimination. Also, *Pf*EMP1 and *Pf*PTP2 are crucial proteins in efficient cell-cell communication between RBCs, reducing inflammatory cytokines and transcriptomic changes in primary human monocytes (71, 72). Recent reports show exosomal microRNAs from RBCs (notably miR-451a and let-7i-5p) differ with sickle trait and malaria status. These RBC-EV-associated miRNAs achieve good discrimination, exosomal miR-451a and let-7i-5p distinguished sickle-cell patients from controls with AUC∼0.83 and correlate inversely with parasite growth. This suggests RBC-EV miR-451a/let-7i can serve as blood biomarkers of SCD genotype and malaria severity (43).

Manakeng et al. reported that chronic inflammation also perturbs RBC-EVs. PS-positive RBC-derived EVs have demonstrated potential in detecting thrombotic events in individuals with systemic lupus erythematosus (SLE). Increased levels of these EVs correlate with previous thrombotic incidents and may signify a heightened risk of vascular problems in SLE (45). PAH patients had significantly more PS-bearing large RBC-EVs, platelets, and medium platelet-derived EVs than normal subjects. Still, they weren’t different from patients who didn’t have PAH (45). The quantity of RBC-EVs in individuals with glomerular haematuria (GH) (renal bleeding) was markedly greater than in those with non-glomerular haematuria (NGH), offering a prospective tool for the classification of GH and a predictive tool to distinguish bleeding sources (47).

Various *in vitro* and *in vivo* studies were carried out to study the role of RBC-EVs in immunomodulation and blood transfusion (73, 74). *In vitro* studies reported that RBC-EVs can interact mostly with monocytes and stimulate the pro-inflammatory cytokines {TNF-α, IL-1, IL-6, and MCP-1, and chemokines [macrophage-derived chemokine (MDC)], and macrophage inflammatory protein 1a, MIP-1a}, contributing to systemic inflammation (75, 76). RBC-EVs, specifically the exosome fraction, increase the ability of APCs to present antigens and stimulate T cells. The increased potency of APCs and the secretion of pro-inflammatory cytokines subsequently boost the proliferation of CD4+ and CD8+ T cells (76). Similarly, during disease conditions like malaria and dengue, RBC-EVs reflect disease burden and immune activation (22). A recent study also reported that RBC-EV treatment significantly reduced inflammation, evidenced by reduced cell death and preservation of retinal function, in a photo-oxidative damage model of retinal degeneration. Multi-omic and *in vitro* analyses indicated that the EVs modulate key pro-inflammatory cytokines implicated in retinal and neurodegenerative disease pathways (73).

Intriguingly, one emerging area is neurodegeneration, RBCs and their constituents exhibit potential as diagnostic instruments in neurodegenerative disorders such as Alzheimer’s disease (AD) and Parkinson’s disease (PD). Research indicates that modifications in red blood cells may signify changes in the brain and could potentially act as indicators for these disorders (77). Studies have explored altered protein kinase C (PKC) conformation and fibril aggregation in AD patient RBCs, suggesting its potential as a diagnostic biomarker, although RBC-EVs require more attention and validation (77, 78). Additionally, reduced alpha-synuclein levels in red blood cells have been noted in Dementia with Lewy Bodies (DLB) relative to healthy individuals and those with Alzheimer’s disease (AD) or Parkinson’s disease (PD), indicating a possible diagnostic differentiator for DLB (79). Although beyond direct RBC measures, RBC-derived exosomes have drawn attention, research highlights the promise of RBC-derived exosomes as non-invasive diagnostics for neurodegenerative diseases, but they need more studies and validations. RBC-EVs in PD patients contain more α-synuclein than healthy subjects, which may play a role in overactivated immunity in monocytes. This suggests RBC-EVs may be useful for early diagnosis and treatment, but the exact link between these changes and PD progression remains unclear (79). Upregulation of miR-125a-5p and downregulation of miR-302a-5p in aged mice are likely associated with neurodegenerative pathways (80).

## 4 Biomarkers in hematologic disorders

RBC-EVs can serve as a potential biomarker for disease dynamics in hematological disorders like ineffective erythropoiesis, hemolysis, and associated severity like sickle cell anemia and thalassemia. For example, in SCD patients, RBC-derived microparticles (RMP) are greatly elevated, which correlates with hemolysis (81). While plasma RMP counts track free hemoglobin, LDH, and bilirubin, and are inversely correlated with Hb and haptoglobin (82). As well, SCD patients with leg ulcers or elevated pulmonary pressures have significantly higher RMP levels, suggesting RBC-EVs mark disease complications (83). Heme-enriched RBC-EVs in SCD patients induce endothelial oxidative stress, adhesion molecule expression, and pro-inflammatory vascular niche, according to a recent study by Giannaki et al. (84). The SCD microRNA study was an observational case-control analysis. Few studies focused on the exosomal miR-451a/let-7i-5p, which showed ∼80–85% sensitivity and specificity for distinguishing SCD genotypes (42, 43). Exosomal miRNAs from RBCs show high diagnostic potential, e.g., in distinguishing sickle cell genotypes (AUC∼0.83). Furthermore, miR-451a is becoming recognized as an important biomarker for red blood cell disorders (8, 68, 69, 71).

In thalassemia, RBC-EVs are similarly enhanced; additionally, β-thalassemia and other chronic anemias induce RBC vesiculation, exhibiting high amounts of RBC-EVs containing oxidized hemoglobin and procoagulant proteins (28). One pilot report also suggests that higher RBC-EV counts may predict thrombotic risk in thalassemia, although formal ROC analysis and diagnostic accuracy metrics have not been established. Additionally, HSP70, HSP90, and peroxiredoxin 6 proteins derived from RBC-EVs were identified as biomarkers for thalassemia and its subtypes (85). Glucose-6-phosphate dehydrogenase (G6PD) deficiency also shows dramatic RBC-EV release, one study found circulating EVs (≈45% RBC-derived) ∼4–10-fold higher in G6PD-deficient versus healthy blood and increasing with disease severity. Thus, elevated RBC-EVs concentration or PS^∧^ + RBC-EVs fraction can distinguish severe hemolytic stages in G6PD deficiency (17, 44). Conversely, alterations in RBC-EVs are less frequently reported in non-hemolytic anemias or bone marrow failure. Previous studies suggest that autoimmune hemolytic anemia or hereditary spherocytosis may enhance RBC vesiculation; however, no extensive clinical research has established their diagnostic significance (53, 86, 87). Overall, RBC-EVs count, or PS positivity, serves as a surrogate marker of ongoing intravascular hemolysis or RBC stress in human studies, even if precise sensitivity/specificity data are not yet defined (66).

## 5 Biomarkers in metabolic conditions

Beyond classic hemolytic/infectious diseases, RBC-EVs show promise in broader conditions. For example, in diabetic cardiovascular disease, co-morbid atrial fibrillation or heart failure is associated with higher RBC-EV levels than diabetes alone, suggesting prognostic value (17). A recent study revealed that RBC-EVs interact with cardiac cells, resulting in modifications to critical signaling pathways, such as cell cycle regulation, proliferation, and oxidative metabolism in a mouse model of ischemic heart disease (88). The concentration of RBC-EVs in patients with type 2 diabetes mellitus (26/μL) exceeded that of patients without type 2 diabetes mellitus (9/μL). Furthermore, the concentration of RBC-EVs, which may serve as a predictor for type 2 diabetes mellitus, exhibited a favorable correlation with fasting blood glucose but not glycated hemoglobin (89).

RBC-EVs also contribute to coagulopathy in hematological disorders, potentially influencing disease progression and patient outcomes. Direct RBC-EV markers in relation to cancer are not yet well established. RBC-EVs profiles may shift due to tumor-induced systemic alterations, although they are generated by bodily cells, they are more abundant in bodily fluids than circulating tumor cells and DNA (90, 91). Cancer-specific miRNAs (miR-103, miR-191, and miR-195) in RBCEVs have been identified as precise biomarkers for distinguishing healthy individuals from breast cancer patients (92). RBC-EVs can also modulate the tumor microenvironment, influence drug resistance, and are being explored as vehicles for targeted drug delivery (93). Their inherited metabolic machinery, immune and mechanical dynamics, plus customizable carrier frameworks, position them as promising diagnostic tools and therapeutic platforms. For instance, engineered RBC-EVs have been proposed as delivery systems for therapeutic agents in chronic lymphocytic leukemia and Burkitt lymphoma (94).

## 6 Clinical significance and limitations

Common RBC-EV markers include (1) EV count or concentration (often CD235a^∧^ + or PS^∧^ + microparticles) and (2) EV cargo molecules (proteins or miRNAs derived from RBCs). Crucially, the specificity and sensitivity of RBC-EV markers vary by context and remain to be fully established (3). However, several studies have quantified RBC-EVs in patient vs. control plasma: e.g., flow-cytometric counts of CD235a^∧^ + EVs or Annexin V^∧^ + EVs, and qPCR assays for RBC-miRNAs in exosomes (95). RBC-EV count (PS^∧^ + microparticles) found elevated in SCD, G6PD deficiency, PAH, SLE, glomerular hematuria, and severe diabetes (15, 17). In contrast, simple RBC-EV counts generally lack defined cutoffs or validated thresholds, but large fold-differences (e.g., 4 × in G6PD) suggest strong sensitivity for distinguishing disease states (17). RBC-EVs can deliver heme to macrophages, too (96). Elevated EV-bound hemoglobin correlates with hemolysis severity, though exact performance data are not defined (53). RBC-EVs naturally display ABO/Rh antigens. In theory, such EVs could be used for non-invasive blood typing, but diagnostics based on these are not yet reported (97). A few clinical validations are underway in specialized research centers, for example, examining RBC-EV profiles in malaria-endemic populations. Nevertheless, no RBC-EV-based test is yet in standard clinical use (25).

Tabular summary of cross disciplinary impact of RBC derived EVs mentioned in Table 3. Diverse methods (ultracentrifugation, size exclusion chromatography, immunoaffinity capture) for the isolation and purification of EVs with variable purity and EV subsets, impairing reproducibility and comparison across studies (98, 99). Moreover, EVs from other hematopoietic or endothelial cells coexist in blood; overlapping size, density, and surface markers (e.g., CD235a for RBCs) complicate accurate RBC-EV quantification (100). RBC-EVs vary by size (exosomes vs microvesicles), content, and trigger for release (oxidative stress, shear stress), challenging biomarker standardization (101). Pre-analytical factors, including sample handling, anticoagulant choice, storage duration, and freeze-thaw cycles, affect EV integrity and yield, leading to inconsistent results (102). Most data also derive from small cohorts or preclinical models; rigorous clinical trials are required to define sensitivity, specificity, and predictive values, hence insufficient large-scale validation (103). Hemolytic conditions, inflammation, infections, or transfusions can elevate RBC-EV levels independently, limiting specificity for malignancies or any pathophysiological conditions (104). Decisively, RBCs are viewed in health and disease and positioned as valuable tools in the future of precision medicine, nanotechnology, and immune modulation (105).

**TABLE 3:** Paradigm shift and future directions.

Domain	Contribution	Implication
Physiology	RBCs modulate immunity, redox balance, and vascular tone	Redefines basic hematology paradigms
Diagnostics	RBC deformability and EVs carry disease signatures	Basis for liquid biopsy and mechanodiagnostics
Therapeutics	RBCs engineered for drug delivery or pathogen trapping	Novel biocompatible treatment platforms
Systems biology	Links between RBCs and circadian rhythms, inflammation	Integrates RBCs into whole-body health regulation

## 7 Conclusion

RBC-derived EVs carry unique cargo, offer a real-time snapshot of ongoing physiological or pathological processes, and can be sampled non-invasively from blood. This makes them ideal for monitoring treatment response or disease progression (e.g., in sickle cell disease flares or response to iron chelation in thalassemia). Elevated RBC-EV levels or RBC-EV-specific contents (like RBC microRNAs) have been reported in anemia, hemoglobinopathies, infections, and inflammatory disorders. Some proposed biomarkers (e.g., exosomal miR-451a) show strong diagnostic accuracy in small studies, while others (RBC-EV counts) require further validation. Their cargo (e.g., heme, Hb, ROS) and surface markers (e.g., PS, CD47) directly modulate endothelial function, trigger immune responses, and amplify thrombo-inflammation, all of which correlate with disease severity and progression. For a translational approach, *in vivo* validation is required for engineered RBCs, RBC-EVs in regenerative medicine, as well as biohybrid micro-devices’ navigation and release control. Overall, current human evidence – especially in plasma/serum samples – suggests RBC-EVs are a rich source of disease biomarkers. Continued clinical research is needed to define their sensitivity/specificity and to translate these findings into validated diagnostic assays.
